# Progression of bone and joint space deformity in patients with mild knee osteoarthritis: Data from the IMI-APPROACH cohort

**DOI:** 10.1016/j.ocarto.2026.100762

**Published:** 2026-03-04

**Authors:** H. Chien Nguyen, Eva Bax, Roel J.H. Custers, Nienke van Egmond, Ruurd J.A. Kuiper, Vahid Arbabi, Hassan Rayegan, Willem Paul Gielis, Ralph J.B. Sakkers, Margreet Kloppenburg, Francisco J. Blanco, Ida K. Haugen, Francis Berenbaum, Mylène P. Jansen, Simon C. Mastbergen, Claudia Lindner, Tim F. Cootes, Harrie Weinans

**Affiliations:** a 3D Lab, University Medical Centre Utrecht, the Netherlands; b Department of Orthopaedic Surgery, University Medical Centre Utrecht, the Netherlands; c Orthopaedic-BioMechanics Research Group, University of Birjand, Iran; d Mechanical Engineering Department, Bozorgmehr University of Qaenat, Iran; e Departments of Rheumatology and Clinical Epidemiology, Leiden University Medical Center, Leiden, the Netherlands; f Servicio de Reumatologia, INIBIC- Hospital Universitario A Coruña, A Coruña, Spain; g Center for Treatment of Rheumatic and Musculoskeletal Diseases (REMEDY), Diakonhjemmet Hospital, Oslo, Norway; h Rheumatology, Sorbonne University, INSERM CRSA, AP-HP Saint-Antoine Hospital Paris, France; i Rheumatology and Clinical Immunology, University Medical Centre Utrecht, the Netherlands; j Division of Informatics, Imaging & Data Sciences, School of Health Sciences, The University of Manchester, UK; k Department Biomechanical Engineering, Faculty 3ME, TU Delft, the Netherlands

## Abstract

**Objective:**

This study aimed to divide leg malalignment into different categories of valgus and varus of the femur, tibia, and intra-articular knee joint and investigates whether knee osteoarthritis (OA) patients are susceptible for changes of such leg deformities over time.

**Design:**

This study included 317 radiographs and CT-images on baseline and 24 months of 169 patients (median age 67, 78.2 % female) of the prospective European IMI-APPROACH cohort, enrolled for knee OA. Femoral, tibial, and intra-articular geometry was determined. Different categories were analysed based on varus or valgus in the femur, in the tibia, or within the intra-articular joint. Changes of these variables over time and their correlations were determined with mixed model analysis.

**Results:**

Femurs tended to become more varus-like over the two-year follow up (0.3°, 95 % CI 0.6°–0.1°, p = 0.02), bony valgus femurs became more varus shaped (1.1°, 95 % CI:1.7°–0.5°, p < 0.001). Patients with bone varus and a normal joint line convergence angle (JLCA) showed a significant increase in intra-articular joint varus, with a mean JLCA increase of 1.1°(95 % CI:0.4°–1.7°, p = 0.005). By two years, they reached the threshold for defining intra-articular joint varus deformity, with a JLCA of 2.0°.

**Conclusions:**

Substantial intra-articular joint and bone varus progression was observed within two years. This study shows that bone deformity is to some extent a dynamic process and there is a growing varus malalignment in the intra-articular knee joint and bones. Thereby this study emphasizes the importance of leg malalignment for progression of intra-articular knee joint changes in early OA.

## 1 Introduction

Osteoarthritis (OA) is a major healthcare burden with an estimated 595 million people worldwide affected in 2020 [1]. The knee is the most affected joint and the disease is characterized as multifactorial, involving structural alterations in the subchondral bone, hyaline cartilage, ligaments, capsule, synovium, and periarticular muscles [2]. The doctrine of OA as a passive wear-and-tear degenerative disease is changing to a much more complex pathogenesis, which involves metabolic, inflammatory, and mechanical factors [2]. Given this complexity, OA healthcare could benefit from more refined disease frameworks derived from phenotypes [3,4] to provide more personalized treatments at an early stage of the disease process.

One of the investigated OA phenotypes is a biomechanically malaligned leg, with a varus or valgus overall leg shape. In general, a deviation of ±2° from neutral hip knee ankle angle (HKA) is considered pathological, although many people with a malalignment are not aware of their deformity [5,6]. Varus and valgus malalignment shift the mechanical leg axis from the middle to respectively the medial or lateral compartment of the knee resulting in potential excessive loading with a higher risk of cartilage and meniscus degeneration in one of these compartments [[2], [3], [4]]. Only radiological progression and not clinical progression has been correlated to malalignment [4,7].

Malalignment of the leg can be the result of three aspects: i) deformity in the tibia; ii) deformity in the femur; and/or iii) non equally distributed cartilage loss in the knee joint, potential surrounding soft tissue laxity, and meniscal abnormalities which leads to an intra-articular joint deformity [6,8].

This study aimed to investigate three potential sources of lower limb deformity, femoral, tibial, and intra-articular knee joint, to assess how bony and intra-articular joint geometries correlate and change over time in patients with knee OA. Our hypotheses is that bone deformities drive intra-articular joint change over time, with valgus bones towards more valgus intra-articular joint progression and varus bones towards more varus intra-articular joing progression.

## 2 Methods

### 2.1 Patients

This study included 317 knee radiographs and whole-body CT-images of 169 patients of the IMI-APPROACH cohort. IMI-APPROACH is a two-year European cohort study to describe, validate, and predict phenotypes of OA using clinical, imaging, and biochemical markers [9]. Recruitment relied on positive clinical American College of Rheumatology criteria for knee OA [9,10] and machine-learning models that guide patient inclusion to predict the probability of increased or sustained knee pain and structural OA progression during the two-years follow-up period. The elaborate selection process and the included patients has been described by van Helvoort et al. [9].

### 2.2 Imaging acquisition

Weight bearing knee radiographs (Buckland-Wright protocol) [9,11] and supine whole-body Computed Tomography (CT) images of 297 patients were available of the index and contralateral side on baseline and after 24 months follow-up, resulting in a total of 594 knees available for analysis [9]. CT images were used for the femoral and tibial analyses of varus and/or valgus. The knee radiographs were used to measure the intra-articular joint varus or valgus.

### 2.3 Image analyses

**Fig. 1:**
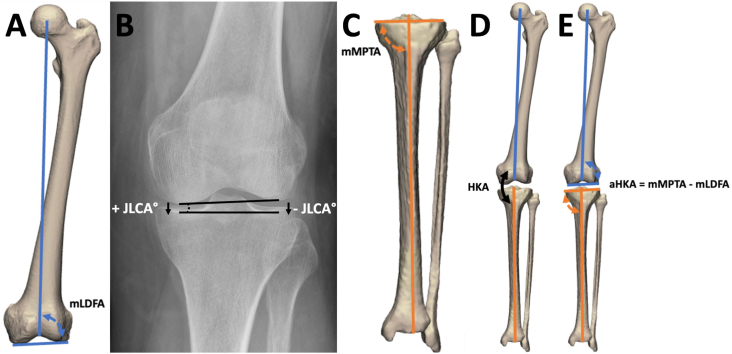
A: Measurement of the mechanical lateral distal femoral angle (mLDFA), B: joint line convergence angle (JLCA), and C: mechanical medial proximal tibial angle (mMPTA). Normal range of mLDFA and mMPTA is between 85° and 90°, while normal range of JLCA is between 0° and 2° [17]. Varus progression has a positive effect on the JLCA and valgus progression a negative effect. D: The hip knee angle (HKA) is measured as the angle between the mechanical axes of the femur and the tibia, E: The arithmetic hip knee ankle angle (aHKA) representing the bony valgus/varus was calculated by subtracting the mLDFA from the mMPTA, a deviation of ±2° from neutral aHKA is considered pathological.

The mechanical lateral distal femoral angle (mLDFA) and mechanical medial proximal tibial angle (mMPTA), both indicators for femoral/tibial varus and/or valgus deformity, were automatically measured on both CT timepoints with validated software (Fig. 1A & C) [12,13]. The intra-articular joint varus or valgus deformity was quantified as the joint line convergence angle (JLCA) on knee radiographs at both time points (Fig. 1B), using the Osteoarthritis Digital Image Analysis (ODIA) [14] tool. Mean error of automated JLCA measurements when compared to manually checked ODIA measurements was defined in a previous study as 0.4° [14].

The hip knee angle (HKA) reflects the amount of varus or valgus in the leg by measuring the angle between the mechanical axes of the femur and tibia (Fig. 1D) on standardized whole leg radiographs [15,16]. The arithmetic hip-knee-ankle angle (aHKA) is a surrogate for the HKA and represents the mechanical axis deviation attributable solely to the bony deformities of the femur and tibia. The aHKA was calculated by subtracting the mLDFA from the mMPTA, resulting in negative values for a bony varus and positive values for a bony valgus (Fig. 1E) [6].

The change over the 24 months follow-up in femoral and tibial geometry was measured for different categories, by dividing the knees into bony and intra-articular space induced deformities based on the JLCA, mLDFA, and mMPTA at baseline (Table 1). The change over 24 months in intra-articular space geometry was also measured for different categories, based on bony and intra-articular space deformities, defined by the JLCA and aHKA on baseline (Table 1). The normal values for mMPTA, mLDFA, aHKA, and JLCA as proposed by Paley were used to differentiate between normal and pathological deformities [6,17]. The change in geometry of the tibia (mMTPA), femur (mLDFA), and intra-articular joint (JLCA) between baseline and 24 months was analysed for each alignment category.

**Table 1:** Selection criteria for the groups’ normal alignment, intra-articular joint valgus or varus deformity, bone valgus or varus deformity, and both bone and intra-articular joint valgus or varus deformity. The selection was based on JLCA measurements on knee radiographs, and mechanical lateral distal femoral angle (mLDFA) mechanical medial proximal tibial angle (mMPTA) on rendered 3D models of the CT-scans. The arithmetic hip knee ankle angle (aHKA) was calculated by subtracting the mLDFA from the mMPTA, representing the bony overall leg alignment.

	JLCA Intra-articular joint varus/valgus	mLDFA Femur varus/valgus	mMPTA Tibia varus/valgus	aHKA Bone varus/valgus
Normal	0°–2*°*	85°–90*°*	85°–90*°*	−2° to 2*°*
JLCA varus	>2*°*	85°–90*°*	85°–90*°*	−2° to 2*°*
Bone varus	0°–2*°*	>90*°*	<85°	< −2°
Bone and JLCA varus	>2*°*	>90*°*	<85°	< −2°
JLCA valgus	<0*°*	85°–90*°*	85°–90*°*	−2° to 2*°*
Bone valgus	0°–2*°*	<85*°*	>90°	>2*°*
Bone and JLCA valgus	<0*°*	<85*°*	>90°	>2*°*
Bone varus and JLCA valgus	<0*°*	>90*°*	<85°	>2*°*
Bone valgus and JLCA varus	>2*°*	<85*°*	>90*°*	< −2°

### 2.4 Statistical analyses

Descriptive statistics were computed using means with the standard deviation (SD) or medians with range, where appropriate. Changes in knee morphology (mMPTA, mLDFA, and JLCA) from baseline to 24 months were analysed using a mixed model analysis, correcting for the possible influence of the inclusion of patients’ left and right knees. The possible difference between changes in the diseased index and contralateral knee was tested using the same mixed model analyses. P values < 0.05 were considered statistically significant. All analyses were performed in IBM SPSS Statistics v26.0.0.1 (Armonk, New York, United States).

## 3 Results

This study included 317 knees of 160 patients with available baseline and 24 months knee radiograph and CT-scan. Table 2 summarizes the baseline and 24 months characteristics of all included knees.

**Table 2:** Baseline and 24 months characteristics of 160 patients with knee osteoarthritis and 317 included knees. BMI = body mass index, KL = Kellgren and Lawrence, mMPTA = mechanical medial proximal tibial angle, mLDFA = mechanical lateral distal femoral angle, JLCA = joint line convergence angle, aHKA = arithmetic hip knee ankle angle.

Knee joint side, n (%)	Left = 160 (*50.5 %*) Right = 157 (*49.5 %*)
Gender, n (%)	Male = 35 (*21.8 %*)
	Female = 125 (*78.2 %*)
BMI (kg/m^2^) at baseline, mean (SD)	27.7 (*SD 5.0*)
Age at baseline, median (range)	67 (*range 48 – 82*)
KL grade of index knee at baseline, n (%)	
Grade 0	84 (*26.5 %*)
Grade 1	94 (*29.7 %*)
Grade 2	67 (*21.1 %*)
Grade 3	62 (*19.6 %*)
Grade 4	10 (*3.2 %*)

	**Baseline**	**24 months**
mMPTA°, mean (SD)	87.0° (*SD 2.1°*)	86.9° (*SD 2.2°*)
mLDFA°, mean (SD)	86.0° (*SD 2.1°*)	86.3° (*SD 2.1°*)
JLCA°, mean (SD)	1.4° (*SD 2.0°*)	1.6° (*SD 2.1°*)
aHKA°, mean (SD)	1.0° (*SD 2.7°*)	0.6° (*SD 2.6°*)

### 3.1 Femoral and tibial varus or valgus changes over time

**Fig. 2:**
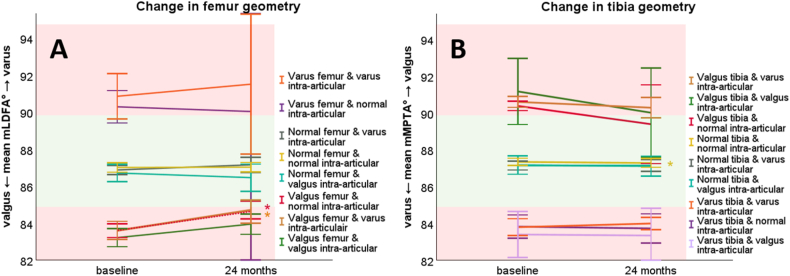
Mean graphs with the 95 % confidence interval (CI) of the change in tibia geometry expressed as mechanical medial proximal tibial angle (mMPTA) and femoral geometry expressed as mechanical lateral distal femoral angle (mLDFA) between baseline and 24 months. The green surface indicates the normal mMPTA and mLDFA value between 85° and 90°, while the red surfaces indicate abnormal mMPTA and mLDFA values. Significant (P ≤ 0.05) changes are marked with “∗” and tested through a mixed model analysis. (For interpretation of the references to color in this figure legend, the reader is referred to the Web version of this article.)

All 317 included femurs tended to become significantly more varus-like (on average 0.3°, 95 % CI 0.6°–0.1°, p = 0.02) over the two years follow-up period. Valgus femurs with normal intra-articular joint geometry (n = 44) had a significant progression towards varus of 1.1° (95 % CI 1.7°–0.5°, p < 0.001) (Fig. 2A, red line). Valgus femurs with intra-articular joint varus (n = 26) revealed a significant progression towards varus too, with on average 1.0° (95 % CI 1.8°–0.3°, p = 0.009) (Fig. 2A, brown line).

Overall, valgus tibias (n = 25) tended to become more varus-like with an average decrease in mMPTA of 0.5° (Fig. 2B, green (*p* = *0.579*), brown (*p* = *0.181*) and red lines (*p* = *0.303*)). This phenomenon was not statistically significant. There was a significant change in mMPTA observed in knees with normal intra-articular joint geometry and mMPTA on baseline (Fig. 2B, yellow line), albeit not relevant as the mean was 0.1° (95 % CI 0.0°–0.2°, p = 0.026) and below the measurement accuracy.

**Fig. 3:**
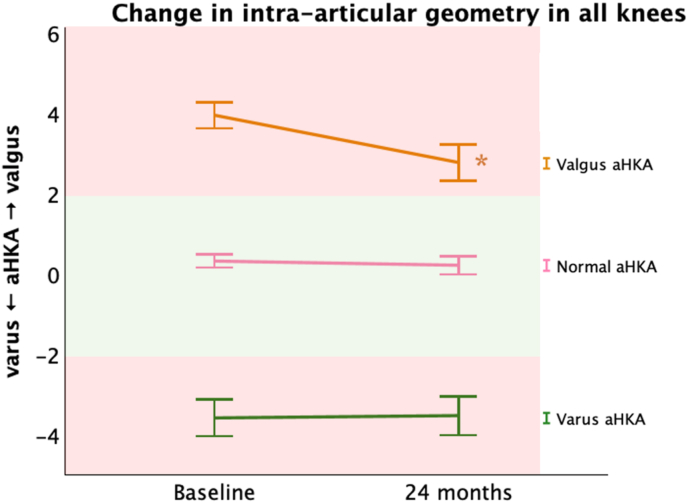
Mean graphs with the 95 % confidence interval (CI) of the change in arithmetic hip-knee-ankle angle (aHKA) between baseline and 24 months. The green surface indicates the normal aHKA value between −2° and 2°, while the red surfaces indicate aHKA values. Significant (P ≤ 0.05) changes are marked with “∗” and tested through a mixed model analysis. (For interpretation of the references to color in this figure legend, the reader is referred to the Web version of this article.)

The combined femur-tibia geometry showed a mean shift toward varus of 1.2° (95 % CI 0.8–1.2°, p < 0.001) in patients presenting with valgus deformity at baseline, indicating progressive varisation of the aHKA over the two-year follow-up (Fig. 3).

There were no significant differences between the index and contralateral knees when analysing the changes in femur and tibia geometry, p = 0.719 for mLDFA and p = 0.746 for mMPTA respectively.

### 3.2 Intra-articular joint varus or valgus changes over time

**Fig. 4:**
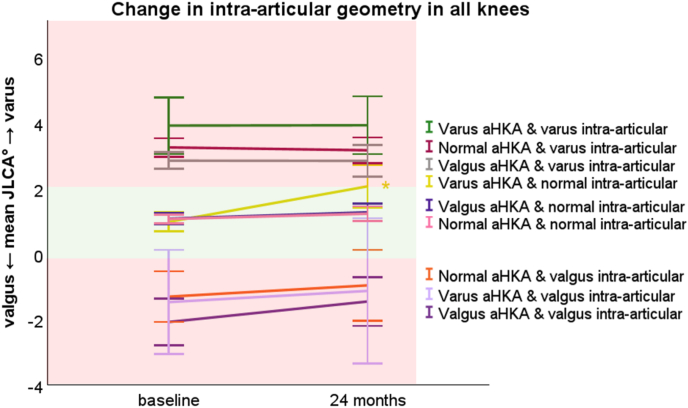
Mean graphs with the 95 % confidence interval (CI) of the change in calculated joint line convergence angle (JLCA) between baseline and 24 months. The green surface indicates the normal JLCA value between 0° and 2°, while the red surfaces indicate abnormal JLCA values. Varus and valgus arithmetic hip knee ankle angle (aHKA) was calculated as surrogate for the bony alignment, by subtracting the mechanical lateral femoral angle from the mechanical medial proximal tibial angle. Significant (P ≤ 0.05) changes are marked with “∗” and tested through a mixed model analysis. (For interpretation of the references to color in this figure legend, the reader is referred to the Web version of this article.)

In general, intra-articular spaces tended to also become more varus like over the two years follow up, meaning an increase in JLCA. Most of these changes towards varus and valgus were non-significant except for the group with bone varus and normal intra-articular joint geometry on baseline. These patients (n = 15) showed a significant increase in intra-articular joint varus after two years, with a mean JLCA increase of 1.1° (95 % CI 0.4°–1.7°, p = 0.005). By two years, they reached the threshold for defining intra-articular joint varus deformity, with a JLCA of 2.0° (Fig. 4, yellow line).

## 4 Discussion

This study aimed to measure the bony and intra-articular joint varus and/or valgus geometry in a knee OA cohort, to classify them into specific categories, and measure the possible changes in geometries over time. Surprisingly, against our expectation, this study observed small changes in femur and tibia geometries even within the two-year follow-up period, with a tendency to become more varus-like during this period. This was mostly pronounced in valgus femurs (on average 1.1°) and valgus tibias (on average 0.5°) on baseline. All intra-articular joint geometries had the tendency to progress towards a more varus shape, except for the knees with an already intra-articular joint varus at baseline. This effect was observed over a relatively short two-year period, and the progression toward varus is expected to continue until it stabilizes. The intra-articular joint progression towards varus was most significant for knees with a normal intra-articular joint geometry and bone varus deformity, which indicates continuous overload at the medial compartment making cartilage tissue susceptible for cartilage degeneration within the medial compartment (Fig. 4 yellow line). Specifically, cartilage degeneration in one knee compartment might further the overall varus or valgus malalignment, thereby creating positive feedback that drives OA progression.

In a recent study by Palmer et al., conducted to explore the potential for clinical and structural progression of OA because of leg malalignment, patient inclusion was determined based on their susceptibility to cartilage degeneration [18]. Patients with joint space widths below 1 mm were excluded, which revealed that varus tibial alignment was associated with increased odds of structural progression in knee osteoarthritis. In agreement, the results of our current study revealed that mainly patients with bone varus and normal intra-articular joint were susceptible for structural radiographical OA progression. The relation between cartilage thickness and change in intra-articular joint geometry was also observed by Colyn et al., with a correlation between Kellgren and Lawrence (KL) grades and JLCA progression [19]. So it seems likely that changes in intra-articular joint geometry are mainly observed in knee joints with mild to moderate OA, and therefore cartilage tissue susceptible for (further) degeneration. Our current study did not include KL grades to test their potential effect on geometry changes over time, which should be considered for future research.

In the study of Colyn et al. the JLCA was measured on standing whole leg radiographs, which also provided the complete varus/valgus stance of the leg. They concluded that the knee progression towards varus was mostly the result of an increased varus shape in the tibia (mMPTA <85°) and increase in intra-articular joint deformity (JLCA >2°) [19]. In our current study, a bony change in both femur and tibia tends to change towards a varus shape. Strangely, this progression happened mostly in the valgus knees and not in the varus knees. Valgus knees may still predominantly load the medial compartment of the knee joint. It should be realized that in normal leg alignment the medial compartment takes significantly more load than the lateral compartment, on average 60 %–70 % of the total load [20]. Hence, even in a slightly valgus shaped leg still most of the loading will pass through the medial side.

Albeit somewhat unexpected, the femoral geometry progression towards varus in valgus knees is worth exploration in future studies. We hypothesize that the change in morphology could possibly be the result of two theories. The first theory comes from gait explorations, in which several studies demonstrated a difference in gait between OA affected and healthy knees [[21], [22], [23]]. This principle of altered gait in patients with knee OA has even been translated into targeted gait alterations as a treatment to achieve pain relief [24]. These alterations in gait support our hypothesis that it is plausible for knee OA patients to develop unique coping mechanisms for pain relief through alterations in their walking patterns. Over time, an altered gait pattern possibly affects the morphology of a bone as a result of different loads. The second theory is that bone morphology only changes at the knee joint ends of the bones due to possible flattening of one compartment and simultaneous formation of subchondral sclerosis and osteophytes because of knee OA. These bony changes in joint ends could potentially affect our calculation of varus and valgus parameters (mLDFA and mMPTA).

Both research and clinical care of knee OA could benefit from further phenotyping of different varus/valgus types as suggested previously [6,25,26]. The result of the current study indicates to differentiate malalignment categories based on the presence of an intra-articular varus/valgus or varus/valgus in the bone structures. The normal range of intra-articular joint geometry is 2° in JLCA, therefore an increase in varus shape of 1.1° within a short period of 2 years is relevant. With this knowledge future studies could attempt to determine more exactly why some patients become progressors, while others are not. This could help in more exact predictions for OA progression and patient indication for therapies.

Patients included in our current study were selected via an algorithm designed by the IMI-APPROACH consortium, based on pain among other things using the ACR criteria [9]. From a surgical perspective, one might justify performing a knee osteotomy in the femur and/or tibia specifically in patients that present a varus malformation. Patient cases presenting knee OA related symptoms and mild radiographical structural manifestations might favour from such an intervention to prevent increases (1.1° in JLCA withing two years) in the overall varus shape, which furthers to progression of cartilage loss in a positive feedback loop. On the contrary, in cases of femoral valgus and normal intra-articular joint geometry a possible delay in surgical intervention might be considered since our data revealed a decrease in valgus shapes even within this relatively short two-year period. The timing of an osteotomy to prevent further OA progression can be challenging, but current data, such as from the IMI-APPROACH cohort, can help guide the optimal timing for this surgery.

Our current study has limitations. First, the formation of osteophytes and bone remodelling due to OA progression could also influence knee geometry measurements, in particular the joint line angles [19,27]. Future research could delve into the possibility of osteophyte formations and the influence on bone geometry measurements. Second, the selection process of the patients led to a skewed distribution between males and females for analyses, since females are predominantly present in the IMI-APPROACH cohort. This potentially led to a more valgus distributed cohort [28]. Third, all measurements were performed fully automatically, which may introduce measurement error. However, this automated method has been extensively validated and shows excellent agreement with manual measurements. Fourth, no clinical outcomes were included in the present study. Future research should evaluate whether changes in leg geometry translate into clinically relevant outcomes. Fifth, the 24 month follow up period may be insufficient to fully capture the natural course of OA progression. Nevertheless, even within this relatively short timeframe, we observed significant changes, indicating that meaningful disease progression can occur over such a period. Sixth, intra-articular joint progression may be influenced by meniscal pathology or meniscectomy, which was not captured in our dataset and should be investigated in future research. This potential contributor should be examined in future studies. Lastly, the IMI-APPROACH cohort included patients based on their likelihood of OA progression. The result of the current study is therefore not representative for healthy patients without clinical symptoms and having varus/valgus deformities.

## 5 Conclusion

Most bones show a trend to become more varus shaped in time, even femurs with a strong valgus shape showed a trend to normalize. Substantial intra-articular joint varus progression was observed within two years, in particular within patients that had bone varus at baseline. This study shows that bone deformity is to some extent a dynamic process and there is a growing varus malalignment in the intra-articular knee joint as well as in the bones. Thereby this study emphasizes the importance of leg malalignment for progression of intra-articular knee joint changes in early OA.

## Authors contributions

Study conception and design: HN, RJB, RC, NE, HW.

Acquisition of data: All authors.

Analysis & interpretation of data: HN, EB, RJB, RC, NE, HW, RK.

Writing of first manuscript draft: HN, EB, RC, NE, RJB, HW.

Critical manuscript revision and approval of final manuscript: All authors.

HN had full access to all of the data in the study and takes responsibility for the integrity of the data and the accuracy of the data analysis.

## Role of the funding source

The funding sources had no role in the design of this study, during its execution, analyses, interpretation of the data, or decision to submit results.

## Declaration of competing interest

H. Chien Nguyen: None.

Eva Bax: None.

Roel J.H. Custers: None.

Nienke van Egmond: None.

Ruurd J.A. Kuiper: None.

Vahid Arbabi: None.

Hassan Rayegan: None.

Willem-Paul Gielis: None.

Ralph J.B. Sakkers: Founder and Advisor Presurgeo BV.

Margreet Kloppenburg: Fee for consulting/advisory boards by Pfizer, UCB, CHDR, GSK, Novartis and Peptinov, all paid to institution. Royalties from Springer Verlag and Wolters Kluwer, all paid to institution. Payment or honoraria for lectures, presentations, speakers bureaus, manuscript writing or educational events from Novartis, paid to institution. Leadership or fiduciary role in other board, society, committee or advocacy group, paid or unpaid at Member OARSI board (2017–2022), Member eular council (chair advocacy committee), President Dutch Society for Rheumatology.

Francisco J. Blanco: Grants or contracts from any entity: Clinical trials of Abbvie, Bristol Myers Squibb, Roche, Servier, Novartis, Horizon Therapeutics Ireland DAC, ITF RESEARCH PHARMA S.L.U., GSK Research, Clinical trials of Pfizer, Sanofi-Aventis, Grunenthal, Lilly, Merck Healthcare KgaA, LG Chem, Ltd, Clinical trials of UCB, Janssen, Amgen, Regeneron, Alkem Laboratories Ltd., Grünenthal, Sun Pharma Global FZE, Kiniksa Pharmaceuticals, GmbH, all paid to institution. Payment or honoraria for lectures, presentations, speakers bureaus, manuscript writing or educational events: Medicamenta-Ecuador, Grunenthal, Asofarma. Support for attending meetings and/or travel: UCB, Abbie, Celgen. Participation on a Data Safety Monitoring Board or Advisory Board: Grunenthal.

Ida K. Haugen: Consulting fees from Novartis, GSK, and Grüntenthal, outside of the submitted work. Payment or honoraria for lectures, presentations, speakers bureaus, manuscript writing or educational events of Abbvie.

Francis Berenbaum: Consulting fees from Grunenthal, GSK, Eli Lilly, Novartis, Pfizer, Servier, 4P Pharma. Honoraria for lectures from Viatris, Pfizer, Zoetis. Support for attending meetings and/or travel from Nordic Pharma. Patents planned, issued or pending for 4Moving Biotech. Participation on a Data Safety Monitoring Board or Advisory Board of AstraZeneca, Sun Pharma, Nordic Bioscience. Stock owner of 4Moving Biotech and 4P Pharma.

Mylène P. Jansen: None.

Simon C. Mastbergen: None.

Claudia Lindner: All support for the present manuscript: Fellowship: Wellcome Trust & Royal Society, UK 223267/Z/21/Z. Patents planned, issued or pending: Patent: Image processing apparatus and method for fitting a deformable shape model to an image using random forest regression voting.

Tim F. Cootes: All support for the present manuscript from EPSRC Funding Body (UK), MRC Funding body (UK), Wellcome Trust, all grant funding to institution. Patents planned, issued or pending: The algorithm for automatically locating points (Random Forest Regression Voting Constrained Local Model) is patented by the University of Manchester.

Harrie Weinans: Founder and Advisor Presurgeo BV.
